# Snake neurotoxin α-bungarotoxin is an antagonist at native GABA_A_ receptors

**DOI:** 10.1016/j.neuropharm.2015.01.001

**Published:** 2015-06

**Authors:** Saad Hannan, Martin Mortensen, Trevor G. Smart

**Affiliations:** Department of Neuroscience, Physiology and Pharmacology, University College London, Gower Street, London WC1E 6BT, United Kingdom

**Keywords:** GABA receptor, Nicotinic acetylcholine receptor, α-bungarotoxin, Dentate gyrus, Electrophysiology, Immunofluorescence, d-Tc, d-tubocurarine, GABA_A_Rs, γ-aminobutyric type-A receptors, α-Bgtx, α-bungarotoxin, nAChRs, nicotinic acetylcholine receptors, α-Bgtx-AF555, α-Bgtx coupled with Alexa Fluor 555, PFA, paraformaldehyde, IPSCs, spontaneous inhibitory postsynaptic currents, DGGCs, dentate gyrus granule cells

## Abstract

The snake neurotoxin α-bungarotoxin (α-Bgtx) is a competitive antagonist at nicotinic acetylcholine receptors (nAChRs) and is widely used to study their function and cell-surface expression. Increasingly, α-Bgtx is also used as an imaging tool for fluorophore-labelling studies, and given the structural conservation within the pentameric ligand-gated ion channel family, we assessed whether α-Bgtx could bind to recombinant and native γ-aminobutyric type-A receptors (GABA_A_Rs). Applying fluorophore-linked α-Bgtx to recombinant αxβ1/2γ2 GABA_A_Rs expressed in HEK-293 cells enabled clear cell-surface labelling of α2β1/2γ2 contrasting with the weaker staining of α1/4β1/2γ2, and no labelling for α3/5/6β1/2γ2. The labelling of α2β2γ2 was abolished by bicuculline, a competitive antagonist at GABA_A_Rs, and by d-tubocurarine (d-Tc), which acts in a similar manner at nAChRs and GABA_A_Rs. Labelling by α-Bgtx was also reduced by GABA, suggesting that the GABA binding site at the receptor β–α subunit interface forms part of the α-Bgtx binding site. Using whole-cell recording, high concentrations of α-Bgtx (20 μM) inhibited GABA-activated currents at all αxβ2γ2 receptors examined, but at lower concentrations (5 μM), α-Bgtx was selective for α2β2γ2. Using α-Bgtx, at low concentrations, permitted the selective inhibition of α2 subunit-containing GABA_A_Rs in hippocampal dentate gyrus granule cells, reducing synaptic current amplitudes without affecting the GABA-mediated tonic current. In conclusion, α-Bgtx can act as an inhibitor at recombinant and native GABA_A_Rs and may be used as a selective tool to inhibit phasic but not tonic currents in the hippocampus.

## Highlights

• Recombinant GABA_A_ receptors are inhibited by α-bungarotoxin
• The β–α subunit interface of GABA_A_ receptors forms the α-bungarotoxin binding site.
• α-bungarotoxin can selectively inhibit α2 subunit-containing GABA_A_ receptors (α2β2γ2).
• α-bungarotoxin inhibits GABA synaptic currents in the hippocampus.
• GABA-mediated tonic currents are unaffected by α-bungarotoxin

## 1 Introduction

The snake venom neurotoxin, α-bungarotoxin (α-Bgtx), binds as an inhibitor with high affinity to nicotinic acetylcholine receptors (nAChRs), including the heteromeric muscle receptors composed of αβγδ or αβδε subunits, and homomeric neuronal subtypes, comprising α7, α8, or α9 subunits (Olsen and Sieghart, 2008; Pirker et al., 2000; Whiting et al., 1995). The γ-aminobutyric acid (GABA) type-A receptors (GABA_A_Rs) are Cl^−^ permeable ligand-gated ion channels from the same pentameric Cys-loop superfamily of receptors as nACh, 5-HT3 and glycine receptors (Smart and Paoletti, 2012). Members of this family share a common structural architecture, including an N-terminal extracellular ligand-binding domain and four α-helical transmembrane-spanning domains (Corringer et al., 2012; Miller and Smart, 2010; Thompson et al., 2010). It has long been of interest that two antagonists at nAChRs, d-tubocurarine (d-Tc; Caputi et al., 2003; Simmonds, 1982; Wotring and Yoon, 1995) and trimethapan (Wotring and Yoon, 1995) are also inhibitors at GABA_A_Rs. In addition, α-Bgtx can also inhibit homomeric β3 subunit-containing GABA_A_Rs by binding at the subunit interfaces (McCann et al., 2006). Whilst it is unclear whether β3 homomers constitute a defined physiological population of GABA_A_Rs, if α-Bgtx can bind at the β3–β3 subunit interface, it is plausible that more physiological αβ subunit-containing GABA_A_Rs (Brickley et al., 1999; Mortensen and Smart, 2006; Sieghart and Sperk, 2002) may be susceptible to block by α-Bgtx by virtue of their β–β subunit interface. By contrast, such an interface should be absent in the more prevalent synaptic-type αβγ GABA_A_Rs (Smart and Paoletti, 2012). We therefore investigated whether non-β3 subunit-containing physiologically-relevant GABA_A_R subtypes can bind α-Bgtx and if so, what are the functional consequences.

By studying recombinant GABA_A_Rs expressed in HEK-293 cells, we reveal that from a selection of αxβ2γ2 heteromers, α-Bgtx inhibited α2β2γ2 receptors to the greatest extent. Furthermore, fluorescent α-Bgtx coupled to Alexa-Fluor 555 (α-Bgtx-AF555) yielded robust staining of α2β2γ2 receptors. This was abolished by d-Tc and by the competitive antagonist at GABA_A_Rs, bicuculline, as well as by GABA, suggesting that the α-Bgtx-binding site on the GABA_A_R heteromers is most probably located at the β–α interface. We also found that α-Bgtx inhibited GABA currents in hippocampal neurons, reducing the amplitudes of synaptic currents. Overall, α-Bgtx is an inhibitor at GABA_A_Rs displaying some selectivity for the α2 subunit-containing isoform.

## 2 Methods

### 2.1 cDNA, plasmids, and drugs

Murine GABA_A_R α1-6, β1-3, γ2 and δ cDNAs subcloned into pRK-5 and pEGFP-C1 have been described previously (Mortensen et al., 2011).

### 2.2 Cell culture and transfection

HEK-293 cells were maintained at 37 °C in a humidified 95% air/5% CO_2_ atmosphere in Dulbecco's modified Eagle's medium (DMEM) supplemented with 10% (v/v) fetal calf serum (FCS), penicillin-G and streptomycin (100 units/ml and 100 μg/ml), 2 mM l-glutamine. Cells were plated onto 22 mm glass coverslips (VWR), coated with poly-l-lysine (Sigma). All media components were from Life Technologies unless otherwise stated.

Cells were transfected with equimolar ratios of cDNAs encoding for α1–6, β1–3 and δ or γ2 GABA_A_ receptor subunits along with eGFP using a calcium phosphate method (Mortensen et al., 2011) 15–45 min after plating.

### 2.3 Primary hippocampal cultures

Dissociated hippocampal neurons were prepared from embryonic day 18 rat pups as described (Hannan et al., 2012). Briefly, hippocampi were dissected in ice-cold Hank's Balanced Salt Solution (HBSS) (Ca^2+^/Mg^2+^ free) before enzymatic dissociation in 0.1% (w/v) trypsin at 37 °C for 10 min followed by serial washes in pre-warmed HBSS to remove trypsin prior to trituration. Cells were mechanically dissociated using fire-polished glass Pasteur pipettes in plating medium composed of minimal essential medium (MEM) supplemented with 5% (v/v) fetal calf serum (FCS), 5% (v/v) horse serum, penicillin-G and streptomycin (200 units/ml and 200 μg/ml), 2 mM l-glutamine and 35 mM glucose. Cells were plated at a density of 10^6^ per ml in plating medium on glass cover-slips previously coated with poly-d-lysine. The neurons were grown and maintained at 37 °C in a humidified 95% air/5% CO_2_ atmosphere.

After plating (30 min), the medium was removed and replaced with maintenance medium composed of Neurobasal-A supplemented with 1% (v/v) B-27, penicillin-G and streptomycin (100 units/ml and 100 μg/ml), 0.5% (v/v) Glutamax and 35 mM glucose.

### 2.4 Preparation of brain slices

Acute transverse brain slices were prepared from adult (P115–125) male C57BL/6J mice in accordance with the UK Animals (Scientific Procedures) Act 1986.

The brain was rapidly removed after terminal anaesthesia with isoflurane and immersed in ice-cold slicing solution composed of (mM): 85 NaCl, 2.5 KCl, 1 CaCl_2_, 4 MgCl_2_, 1.25 NaH_2_PO_4_, 26 NaHCO_3_, 75 sucrose, and 25 glucose, 2 kynurenic acid, pH – 7.4. The slicing solution was continuously bubbled with 95% air and 5% CO_2_. Transverse 250 μm slices containing the ventral hippocampus were cut with a Leica VT1200S vibroslicer. The slicing solution was exchanged at 37 °C for 60 min with a recording solution containing (mM): 125 NaCl, 2.5 KCl, 2 CaCl_2_, 1 MgCl_2_, 1.25 NaH_2_PO_4_, 26 NaHCO_3_, 2 kynurenic acid, and 25 glucose, pH 7.4.

### 2.5 Whole-cell patch-clamp electrophysiology

Whole-cell GABA-activated currents were recorded from transfected HEK-293 cells or hippocampal neurons in culture at 12–14 DIV using patch clamp electrophysiology. Patch electrodes had resistances of 4–5 MΩ and were filled with an internal solution containing (mM): 120 CsCl 1 MgCl_2_, 11 EGTA, 30 KOH, 10 HEPES, 1 CaCl_2_, and 2 K_2_ATP; pH – 7.2. HEK-293 cells were superfused with a saline solution containing (mM): 140 NaCl, 4.7 KCl, 1.2 MgCl_2_, 2.52 CaCl_2_, 11 Glucose, and 5 HEPES; pH 7.4. The saline for recording from primary neurons was supplemented with 2 mM kynurenic acid and pH adjusted to 7.4 to block all spontaneous excitatory post-synaptic currents (EPSCs). Membrane currents were filtered at 5 kHz (−3 dB, 6th pole Bessel, 36 dB/octave). HEK-293 cells were studied 48 h after transfection by voltage clamping cells at a holding potential of −20 to −40 mV with optimised series resistance (Rs, <10 MΩ) and whole-cell membrane capacitance compensation. Neuronal membrane currents were similarly recorded at a holding potential of −60 mV. Changes of Rs greater than 10% during the experiment resulted in the recording being excluded from analysis.

GABA concentration–response curves were generated by measuring the current (I) at each GABA concentration, applied at suitable time intervals, and normalizing the current to the maximum GABA response (I_max_), before fitting the concentration response relationship with:

$$\text{I}/{\text{I}}_{\max}=\left[\left(1/1\right)+{\left({\text{EC}}_{50}/\text{A}\right)}^{n}\right]$$

where A is the concentration of GABA, EC_50_ is the concentration of GABA giving 50% of the maximum response and n is the Hill slope.

For studying inhibition, α-Bgtx was either co-applied, or pre-applied for 30–60 s, followed by co-application with sub-maximal doses of GABA. Spontaneous inhibitory postsynaptic currents (IPSCs) were recorded from dentate gyrus granule cells (DGGCs) with the same internal solution as above. Cells were voltage clamped at −60 mV and IPSCs were recorded using 5 kHz filtering with optimal series resistance and whole-cell capacitance compensation.

IPSCs were detected and analysed using WinEDR and WinWCP (John Dempster, University of Strathclyde, UK). IPSC frequency was calculated using events detected over 60 s epochs of recording. For IPSC amplitudes, in excess of several thousand IPSCs were recorded and analysed as overall mean and then displayed as an amplitude distribution and fitted with a sum of 1–4 Gaussian functions of the form:

$$f\left(x\right)=A\frac{e^{-{\left(x-\mu \right)}^{2}/2\sigma i^{2}}}{\sigma \sqrt{2\pi }}+C$$

Where *A* defines the amplitude and *C* is a constant defining the pedestal of the histogram. This function provided the Gaussian mean amplitude current (μ) and standard deviation (σ). All the distributions were fitted using this function in Origin (Ver 6). The accuracy of the fits was checked by repeating the iterative non-linear fitting procedure after substituting the best-fit parameters obtained for the control and α-Bgtx datasets with new values.

For tonic inhibition, to determine the average holding currents, a 60 s continuous current recording was sampled every 1 s, discarding epochs that coincided with IPSCs. Any effect of drugs on the holding current was defined by subtracting the average holding currents in control and during drug application.

The baseline noise (RMS) was calculated before and during drug treatment. This was estimated from a continuous (30 s) current recording, sampled every 100 ms. The median current was calculated every 5 s and values more than twice the standard deviation from the median (usually due to IPSCs) were eliminated. Baseline GABA-mediated current noise was defined by subtracting RMS values before and after drugs, e.g., α-Bgtx or bicuculline.

### 2.6 Fluorescent α-Bgtx staining and imaging

Live transfected HEK-293 cells were studied 48 h after transfection and washed with Krebs to remove cell culture media and incubated in 400 nM α-Bgtx coupled with Alexa Fluor 555 (α-Bgtx-AF555; Life Technologies) for 10 min at room temperature (RT). Cells were washed and fixed in 4% paraformaldehyde (PFA; Sigma) for 10 min at RT. The cells were imaged immediately post-fixation in saline using a Zeiss LSM 510 Meta confocal microscope and an Achroplan x40 water DIC objective (NA 0.8) as described previously (Hannan et al., 2012). This involved choosing the optimal z-section and acquiring images as a mean of 4 scans in 16-bits using a 543 nm Helium–Neon laser and a 560 nm long-pass filter for α-Bgtx-AF555 and a 488 Argon laser with a 505–530 nm band-pass filter for eGFP.

In experiments using permeabilisation, cells were fixed in 4% PFA for 10 min at RT followed by washes (x3) in phosphate buffered saline (PBS; Sigma) and 0.1% triton-X100 (Sigma) was added for 10 min at RT in 10% (v/v) FCS. Cells were washed to remove the detergent and 400 nM α-Bgtx-AF555 was added for 10 min at RT to label intracellular receptors.

### 2.7 Image analysis

Confocal images were analysed using ImageJ (version 1.410) as described previously (Hannan et al., 2013). For each cell the surface membrane was identified by drawing a region-of-interest (ROI) in the eGFP channel and this was transferred to the α-Bgtx-AF555 channel and the mean membrane fluorescence values were determined. Mean background fluorescence was determined from a region devoid of cells. This was subtracted from the mean membrane fluorescence providing a mean corrected fluorescence intensity value. These values, for different combinations of receptors and drugs, were graphically plotted using Origin.

## 3 Results

### 3.1 Bungarotoxin inhibits GABA_A_ receptors in hippocampal neurons

Heteromeric αβγ receptors are a predominant GABA_A_R subtype in the neocortex, including the hippocampus (Olsen and Sieghart, 2008; Pirker et al., 2000; Whiting et al., 1995). A smaller proportion of receptors in these areas are thought to be αβ heteromers, but to date, there is little if any direct evidence to support the existence of β3 homomers in neurons (Mortensen and Smart, 2006). Before commencing recombinant receptor studies, we first examined α-Bgtx and two other nAChR antagonists for their ability to inhibit whole-cell GABA-activated currents in primary hippocampal neurons, which express heteromeric GABA_A_Rs.

**Fig. 1:**
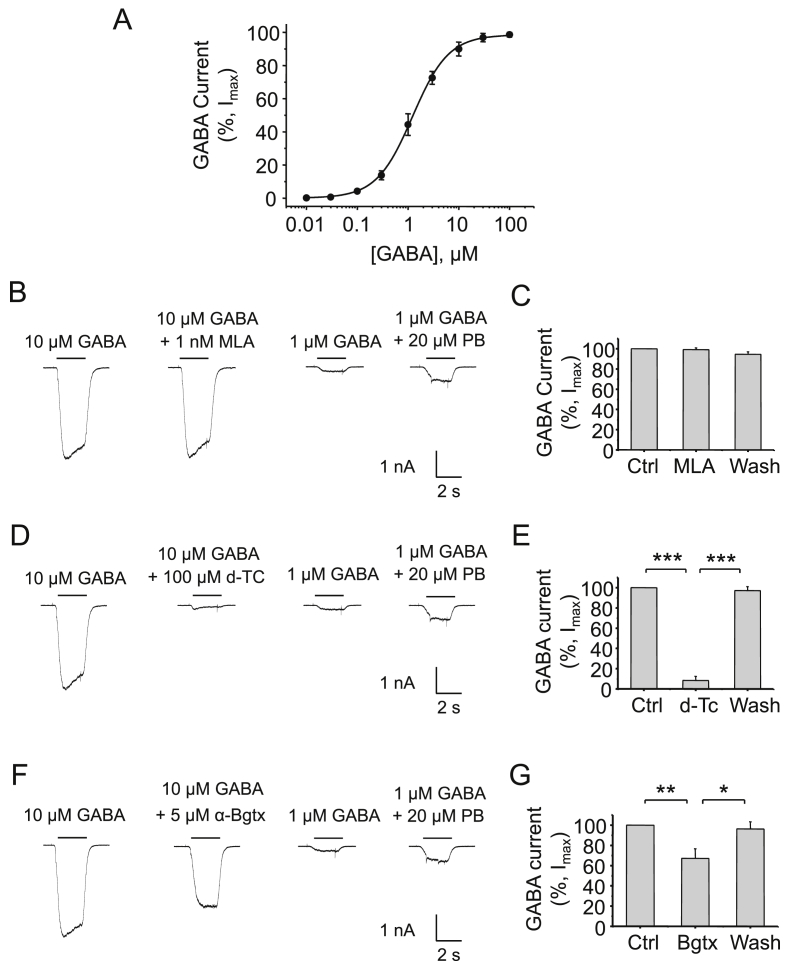
**Inhibition of native hippocampal GABA**_**A**_**Rs by d-Tc and α-Bgtx**. A, GABA concentration response curve from primary hippocampal neurons. Whole-cell GABA-activated currents recorded from rat hippocampal neurons in culture (12–14 DIV) in response to 10 μM GABA (left hand panels) and GABA + 1 nM MLA (B,C); +100 μM d-tubocurarine (d-Tc) (D,E); +5 μM α-Bgtx and 1 nM MLA (F,G). Example control traces also show the potentiation of 1 μM GABA currents with 20 μM pentobarbital. MLA and or α-Bgtx were pre-applied for 1 min before co-application with GABA. Bargraph data presented here and in succeeding figures are means ± S.E.M, n = 5–8 cells; *P < 0.05, **P < 0.01, ***P < 0.001; One-way ANOVA.

Receptors were activated by GABA (EC_50_ = 1.23 ± 0.04 μM; n = 10; Fig. 1A) in the presence of 2 mM kynurenic acid to block excitatory postsynaptic currents (EPSCs). The potent α7 nAChR specific antagonist methyllaconitine (MLA) did not affect currents activated by sub-maximal GABA (10 μM) concentrations, either when co-applied with GABA (<1% of control; data not shown) or when co-applied with GABA after a 1 min pre-incubation with 1 nM MLA (0.8 ± 1.7% inhibition; n = 6; P > 0.05; Fig. 1B–C) indicating that MLA is not an antagonist at GABA_A_Rs.

The non-selective nAChR antagonist, d-tubocurarine (d-Tc), is a known competitive antagonist at GABA_A_Rs (Simmonds, 1986) in the cuneate nucleus (Simmonds, 1982), substantia nigra (Caputi et al., 2003), and hippocampus (Lebeda et al., 1982; Wotring and Yoon, 1995). As expected, 100 μM d-Tc substantially inhibited whole-cell GABA-activated currents in cultured hippocampal neurons (92 ± 4% inhibition; n = 5; P < 0.001, Fig. 1D–E).

We next introduced α-Bgtx, a non-selective competitive nAChR antagonist. Pre-applying 5 μM α-Bgtx to cultured hippocampal neurons for 1 min in the presence of 1 nM MLA (to inhibit any crosstalk with endogenous nAChRs) followed by co-application with 10 μM GABA resulted in significant inhibition of GABA currents (by 33 ± 9%; n = 8; P < 0.01; Fig. 1F–G). The effect of α-Bgtx was reversible with GABA currents returning to control levels after 60 s recovery (Fig. 1G; P < 0.05). As a control, these receptors were also modulated by pentobarbital with 1 μM GABA currents being potentiated by 20 μM pentobarbital in the same neurons (Fig. 1B, D, F).

These data indicated that α-Bgtx can antagonise GABA-activated currents in neurons, but the level of inhibition was surprisingly high given that we would expect only a very small proportion, if any, of native GABA_A_Rs to contain a β3–β3 interface. These results therefore suggested that α-Bgtx may be an antagonist at native heteromeric GABA_A_Rs, and possibly target a binding site that is discrete from the β–β subunit interface.

### 3.2 Bungarotoxin binds to a subset of recombinant GABA_A_R heteromers

Having established that α-Bgtx is an antagonist at native GABA_A_Rs, we explored its ability to bind to recombinant GABA_A_R subtypes considered to be physiologically relevant. To avoid any confounds, we did not include β3 subunits because of the potential to form β3–β3 interfaces which would bind α-Bgtx as previously reported (McCann et al., 2006).

**Fig. 2:**
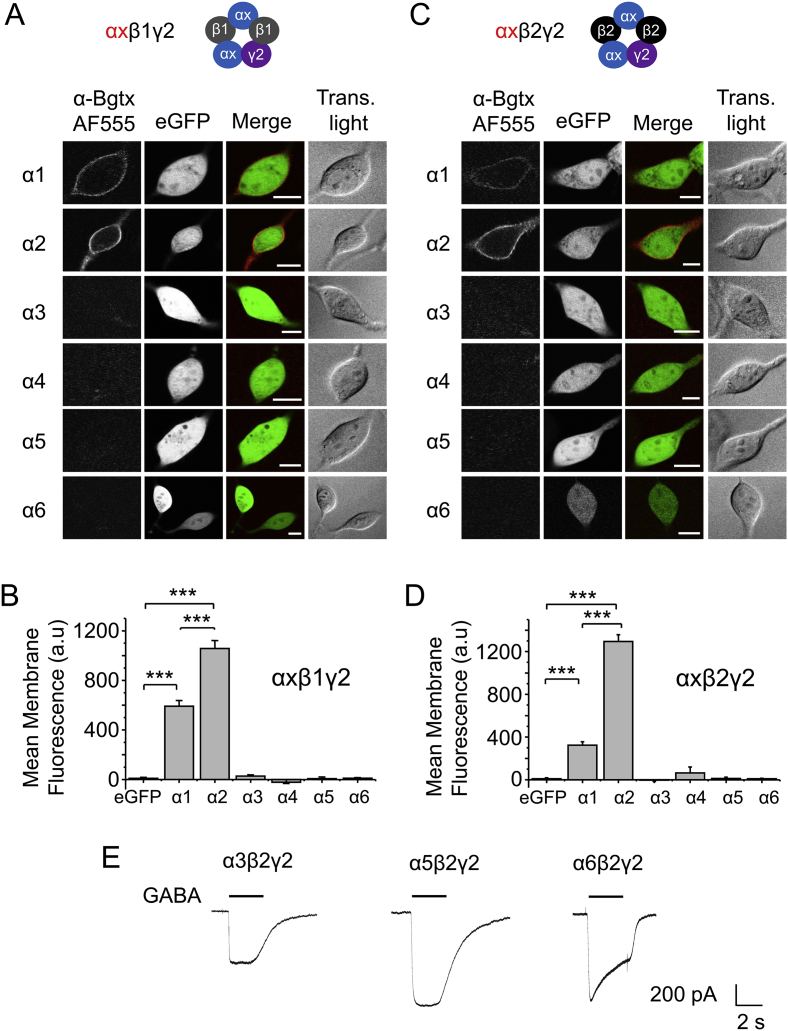
**GABA**_**A**_**R heteromers bind to Alexa Fluor 555-labelled α-Bgtx**. HEK-293 cells expressing eGFP, α1-6 and γ2 with either β1 (A) or β2 (C) subunits, were incubated in 400 nM α-Bgtx coupled to Alexa Fluor 555 (α-BgTx-AF555), 48 h post-transfection, for 10 min at RT. Cells were washed to remove excess α-Bgtx-AF555, fixed and imaged. (B,D) Mean cell surface fluorescence of α-Bgtx-AF555 bound to GABA_A_Rs expressing eGFP alone, or eGFP and α1-6β1γ2 (B) or α1-6β2γ2 (D). ***P < 0.001, n = 6–9 cells; Scale bar 5 μm. Example traces of whole-cell 10 μM GABA-activated currents recorded from HEK cells expressing α3/5/6β2γ2 demonstrate the functional expression of these receptors.

We first investigated the binding of α-Bgtx coupled to Alexa-Fluor 555 (α-Bgtx-AF555) to cell surface receptors expressed in HEK-293 cells composed of an α subunit (α1-α6) with either β1γ2 (Fig. 2A–B) or β2γ2 (Fig. 2C–D) subunits. Of these combinations, α2 subunit-containing receptors demonstrated the highest cell surface staining with α-BgTx-AF555 (Fig. 2A, C). The mean cell surface fluorescence of cells expressing α2β1γ2 (fluorescence intensity = 1057 ± 64 a.u., n = 6; Fig. 2A–B) and α2β2γ2 (fluorescence intensity = 1294 a.u., n = 6; Fig. 2C–D) were significantly higher compared to cells expressing only eGFP (fluorescence intensity = 9 ± 4 a.u., n = 6; P < 0.001; one way ANOVA; Fig. 2B, D).

Weaker staining intensities were measured for α-Bgtx-AF555 binding to other receptor isoforms: α1β1γ2 (592 ± 46 a.u., n = 6; Fig. 2A–B), α1β2γ2 (324 ± 33 a.u., n = 6; Fig. 2C–D), α4β1γ2 (90 ± 64 a.u., n = 6; Fig. 2A, C), and α4β2γ2 (64 ± 56 a.u., n = 6; Fig. 2B, D). These staining intensities for α1β1/2γ2, but not for α4β1/2γ2, were significantly greater compared to eGFP alone (P < 0.001). Receptors containing α3/5/6 subunits and β1/2γ2 did not show any labelling with α-BgTx-AF555 (P > 0.05). The imaging data demonstrated that α-Bgtx binds to several combinations of physiologically important heteromeric GABA_A_Rs in HEK-293 cells and that binding does not require the presence of one or more β3 subunits.

To discount the possibility that the failure of α-Bgtx to bind to receptors containing α3/5/6 subunits was due to poor receptor expression, we used patch clamp recording to examine their responsiveness to GABA. Expressing α3/5/6 subunits with β2γ2 subunits produced receptors that supported robust GABA currents (Fig 2E) confirming that the lack of staining observed with α-Bgtx for these receptors was not due to a lack of expression.

### 3.3 Bungarotoxin selectively inhibits α2 subunit-containing receptors

To complement our imaging studies we next examined the effect of α-Bgtx on GABA_A_R function using whole-cell patch electrophysiology in HEK-293 cells expressing αxβ2γ2 receptor subtypes. We selected the following subunit combinations based on their relative abundance in the hippocampal *stratum pyramidale*: α1β2γ2 and α2β2γ2, reflecting their relative importance as non-β3-containing synaptic GABA_A_Rs; α4β2γ2 and α5β2γ2, chosen since they may represent forms of extrasynaptic GABA_A_Rs in the hippocampus that underpin tonic inhibition (Glykys et al., 2008).

**Fig. 3:**
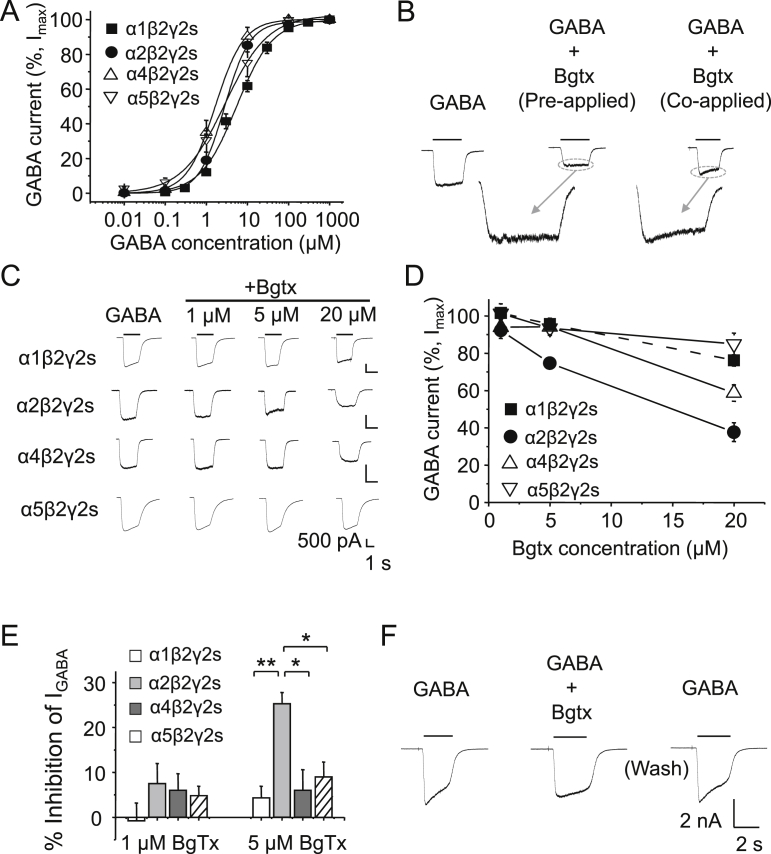
**α-Bgtx inhibition at GABA**_**A**_**Rs expressed in HEK293 cells**. A, GABA concentration response curves for α1β2γ2, α2β2γ2, α4β2γ2, and α5β2γ2 receptors expressed in HEK-293 cells. B, GABA current profiles for α4β2γ2 receptors in response to 3 μM GABA, and +20 μM α-Bgtx (with or without pre-application for 1 min). Inserts show expanded current profiles. C, Representative whole-cell GABA-activated currents in response to submaximal concentrations of GABA in the absence (left-panels) or presence of 1, 5 and 20 μM α-Bgtx (pre-applied for 1 min) for cells expressing α1β2γ2, α2β2γ2, α4β2γ2 and α5β2γ2 receptors. D, Inhibition of GABA-activated currents by α-Bgtx. GABA concentrations are 6 μM (α1β2γ2), and 3 μM (α2β2γ2, α4β2γ2, and α5β2γ2). Lines are drawn (n = 3). E, Inhibition at receptors by 1 and 5 μM α-Bgtx. With 5 μM α-Bgtx, the inhibition observed at α2β2γ2 was statistically significant compared to α1β2γ2 (**P < 0.01), and α4β2γ2 and α5β2γ2 (*P < 0.05) receptors, n = 3, One-way ANOVA. F, Representative whole-cell GABA-activated currents in response to maximal concentration of GABA (1 mM) in the presence of 5 μM α-Bgtx and after recovery from inhibition.

The ability of 1, 5 or 20 μM α-Bgtx to inhibit submaximal GABA currents was examined. The GABA concentrations used were 6 μM for α1β2γ2, and 3 μM for α2β2γ2, α4β2γ2, and α5β2γ2, based on their pre-determined GABA EC_50_s of: 6.6 μM (α1β2γ2; pEC_50_ 5.18 ± 0.06, n = 34); 2.76 μM (α2β2γ2; pEC_50_ 5.46 ± 0.09, n = 5); 1.68 μM (α4β2γ2; pEC_50_ 5.78 ± 0.05, n = 5); and 2.44 μM (α5β2γ2; pEC_50_ 5.61 ± 0.24, n = 5; Fig. 3A).

For these receptor subtypes there were notable differences in the GABA current profiles as expected from their subunit composition (Mortensen et al., 2011; Picton and Fisher, 2007). Differences in GABA current profiles were also observed depending on whether α-Bgtx was co-applied with GABA or also pre-applied for 1 min (Fig. 3B). The level of block was increased by pre-application of α-Bgtx and the slow sag in the GABA current, evident from just co-applying α-Bgtx, suggested that the toxin binds to GABA_A_Rs with a slow on-binding rate. Therefore, to achieve a full steady-state block, α-Bgtx was pre-applied.

Using this protocol, GABA currents at all GABA_A_R subtypes examined were inhibited by the highest concentration of α-Bgtx tested (20 μM), with currents mediated by α2β2γ2 showing the most inhibition, and α5β2γ2 the least. The level of inhibition at 20 μM α-Bgtx in ascending order (Fig. 3C–D) is: 15.3 ± 5.9% (α5β2γ2), 23.8 ± 3% (α1β2γ2), 41.3 ± 4.4% (α4β2γ2), and 62.3 ± 5% (α2β2γ2) (n = 3).

At lower concentrations of α-BgTx (5 μM) however, inhibition was only observed at α2β2γ2 receptors (Fig. 3C–E). These results are consistent with our data from fluorescent α-BgTx labelling with α2β2γ2 and correlates the highest levels of staining with a low concentration (400 nM) of α-BgTx-AF555 (Fig. 2) with the highest sensitivity to block by α-Bgtx (Fig. 3D–E).

To examine the nature of α-Bgtx inhibition at GABA_A_Rs, we applied 5 μM α-BgTx with 1 mM GABA, to study antagonism at saturating GABA concentrations. Saturating GABA currents were reduced by 13.0 ± 5.9% (n = 3) in the presence of α-BgTx indicative of some mixed-non competitive antagonism (Fig. 3F).

### 3.4 β-α subunit interface forms a bungarotoxin-binding site in GABA_A_Rs

**Fig. 4:**
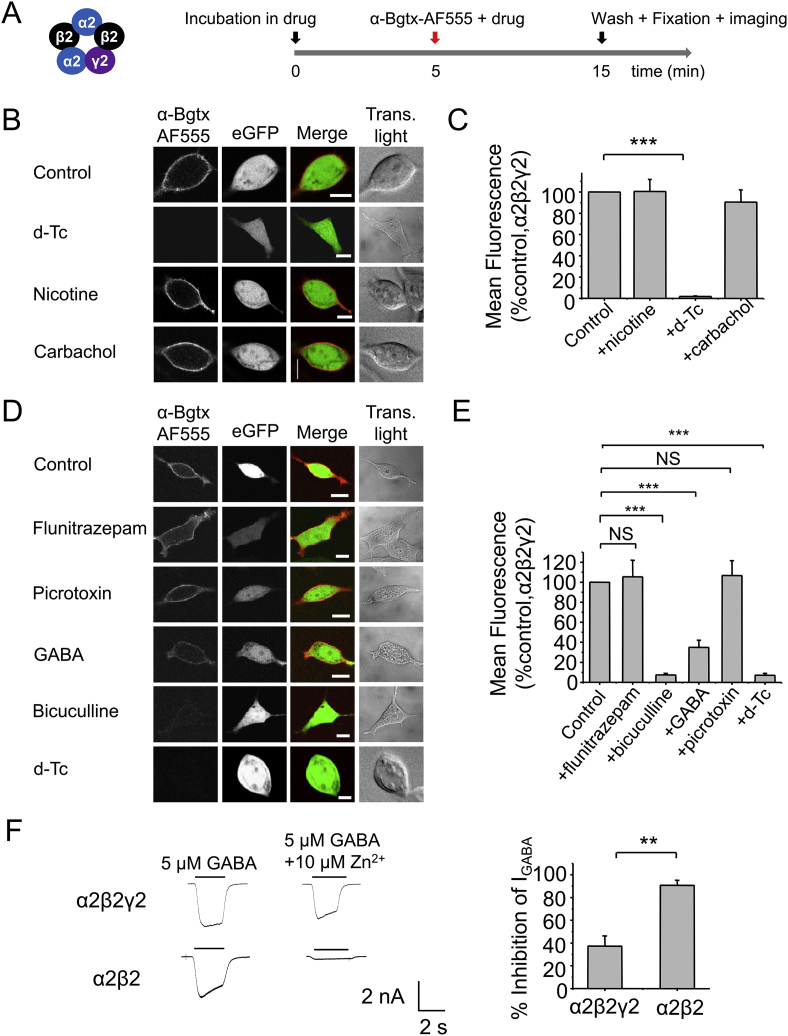
**α-Bgtx binds at the β–α subunit interface of GABA**_**A**_**Rs**. A, Schematic of the experimental protocol. 48 hrs after transfection, HEK-293 cells expressing eGFP and α2β2γ2 were incubated in drug for 5 min at RT followed by the addition of drug +400 nM α-Bgtx-AF555 for 10 min at RT to label cell surface receptors. The cells were washed to remove the excess α-Bgtx-AF555, fixed and imaged. B, Images of cells expressing α2β2γ2, stained with α-Bgtx-AF555 in the presence of 1 mM d-Tc, 1 mM nicotine, or 1 mM carbachol. C, Mean cell surface fluorescence of α-Bgtx-AF555 bound to surface GABA_A_Rs in the presence of d-Tc, nicotine or carbachol. D, Images of cells expressing α2β2γ2, stained with α-Bgtx-AF555 in the presence of 250 μM GABA, 50 μM bicuculline, 1 mM d-Tc, 500 nM flunitrazepam or 20 μM picrotoxin. E, Mean cell surface fluorescence of α-Bgtx-AF555 bound to GABA_A_Rs in the presence of: GABA, bicuculline, d-Tc, flunitrazepam, picrotoxin. F, 5 μM GABA-activated currents and mean (±sem) inhibition caused by 5 μM Zn^2+^ for HEK-293 cells expressing α2β2γ2 or α2β2 receptors, 48 h post-transfection. ***P < 0.001, **P < 0.01; n = 6–7. Scale bars 5 μm.

To identify the location of the α-Bgtx binding site on GABA_A_Rs, we pre-incubated HEK-293 cells expressing α2β2γ2 with a range of ligands that act at GABA_A_Rs and nAChRs, for 5 min at RT followed by co-incubation with α-Bgtx-AF555 for 10 min at RT (Fig. 4A). These ligands were selected to ‘protect by binding occupancy’ known binding site domains on GABA_A_Rs and nAChRs.

We first studied the effect of some well-characterised nAChR ligands on α-Bgtx-AF555 binding to α2β2γ2 GABA_A_Rs on the assumption that a similar α-Bgtx-binding site may exist on both receptor types. Neither of the nAChR agonists, 1 mM nicotine (90.5 ± 11.4% of control, P > 0.05, one-way ANOVA) nor 1 mM carbachol (100.5 ± 11.3% of control, P > 0.05), had any effect on the binding of α-Bgtx-AF555 to α2β2γ2 (n = 7, Fig. 4B–C); however, the antagonist, d-Tc (1 mM) abolished α-Bgtx-AF555 binding (1.8 ± 1.3% of control, n = 7; Fig. 4B–C; P < 0.001).

As d-Tc is a competitive antagonist at GABA_A_Rs (Wotring and Yoon, 1995), the abolition of α-Bgtx-AF555 staining suggested that the GABA-binding β–α interface may form the α-Bgtx-binding site on α2β2γ2 heteromers. In HEK-293 cells, transfected α2, β2, and γ2 subunits are thought to assemble with a subunit order of β2-α2-β2-α2-γ2, and therefore, β2-β2 interfaces should be absent (Smart and Paoletti, 2012).

We next studied the effects of selective ligands for GABA_A_Rs on α-Bgtx-AF555 binding to α2β2γ2. The benzodiazepine (BDZ) flunitrazepam (500 nM), which potentiates GABA_A_R currents by binding at the α-γ interface (Pritchett et al., 1989; Richter et al., 2012; Sigel, 2002) did not affect α-Bgtx-AF555 binding to α2β2γ2 (105. 5 ± 16.5% of control, n = 6; Fig. 4D–E; P > 0.05, One-way ANOVA). The non-competitive GABA channel blocker, picrotoxin (20 μM), also had no effect on α-Bgtx-AF555 binding (106.7 ± 14.9% of control, n = 6; Fig. 4D–E; P > 0.05). These results indicated that the α-γ interface and deep within the ion channel pore are unlikely sites for α-Bgtx binding to GABA_A_Rs.

We then explored the β–α interfaces which form the GABA binding sites in GABA_A_Rs (Sieghart, 1995). GABA (250 μM) reduced α-Bgtx-AF555 binding significantly (34.9 ± 7.1% of control, n = 6; Fig. 4D–E; P < 0.001); and the competitive antagonists, bicuculline (50 μM) (7. 5 ± 1.5% of control, n = 6; Fig. 4D–E; P < 0.001) and d-Tc (1 mM) (7.1 ± 1.9% of control, n = 6; Fig. 4D–E; P < 0.001), virtually eliminated α- Bgtx-AF555 binding to α2β2γ2, supporting a role for the β–α interface in binding α-Bgtx to GABA_A_Rs.

To ascertain whether our αβγ receptors were assembled intact ensuring the absence of β–β subunit interfaces, we studied the inhibition caused by Zn^2+^ at α2β2γ2 receptors. Receptors composed of αβ subunits will be inhibited significantly more by Zn^2+^ when compared to αβγ receptors (Hosie et al., 2003; Krishek et al., 1998). Consistent with this, we found that 10 μM Zn^2+^ significantly inhibited submaximal (5 μM) GABA currents for α2β2 by 90.8 ± 4.44% (n = 3; Fig. 4F; P < 0.01) compared to 37.4 ± 8.9 for α2β2γ2 (n = 3) suggesting that most of the receptors used in the imaging studies are likely to contain γ2 subunits. Furthermore, given that our results so far suggest that α-Bgtx binds to the β–α interface, the incorporation of the γ2 subunit is unlikely to be the determining factor for α-Bgtx binding to GABA_A_Rs.

**Fig. 5:**
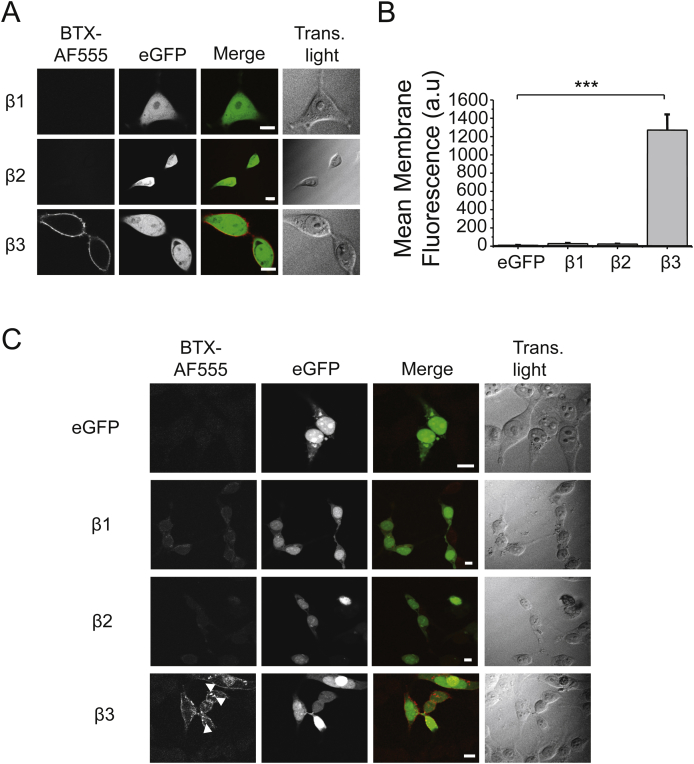
**α-Bgtx-AF555 binds to β3 but not β1 or β2 subunits**. A, Images of HEK-293 cells expressing eGFP with either β1, β2 or β3 subunits, incubated with 400 nM α-Bgtx-AF555 for 10 min at RT, 48 h after transfection, washed to remove the excess α-Bgtx-AF555, fixed and imaged. B, Mean surface membrane fluorescence of cells expressing eGFP and β1–3 subunits. ***P < 0.001, n = 6–9. C, Images of HEK-293 cells expressing eGFP with or without either β1, β2 or β3 subunits, after fixation in 4% PFA, 48 h after transfection, and permeabilised with 0.1% w/v Triton-X100, and incubated in 400 nM α-Bgtx-AF555 for 10 min at RT, washed and imaged. Arrowheads indicate intracellular structures labelled with α-Bgtx-AF555. Scale bars 5 μm (A) and 10 μm (C).

Given that the β3–β3 interface forms an α-Bgtx binding site, and that GABA receptor β subunits are highly homologous, we investigated whether the β1–β1 or β2-β2 interfaces could also form α-Bgtx binding sites. However, for cells expressing β1, β2 or β3 homomers in HEK-293 cells for 48 h and incubated in α-Bgtx-AF555 with or without permeabilisation, only β3 expressing cells showed high levels of cell surface and intracellular staining with α-Bgtx-AF555 (Fig. 5A–C). Such staining was absent for β1 and β2 subunits discounting the possibility that β–β subunit interfaces were the sites for α-Bgtx binding in β1 or β2 subunit-containing heteromeric GABA_A_Rs.

### 3.5 Bungarotoxin inhibits only phasic GABA currents in dentate gyrus granule cells

Having established the selectivity of micromolar α-Bgtx concentrations for inhibiting α2β2γ2 receptors, we assessed the sensitivity of native GABA_A_Rs in adult (P115–125) mouse acute hippocampal slices to nAChR ligands and to α-Bgtx. Voltage-clamp recordings of spontaneous inhibitory postsynaptic currents (IPSCs) were performed in dentate gyrus granule cells (DGGCs), which express α2, β2 and γ2 subunits, amongst others.

**Fig. 6:**
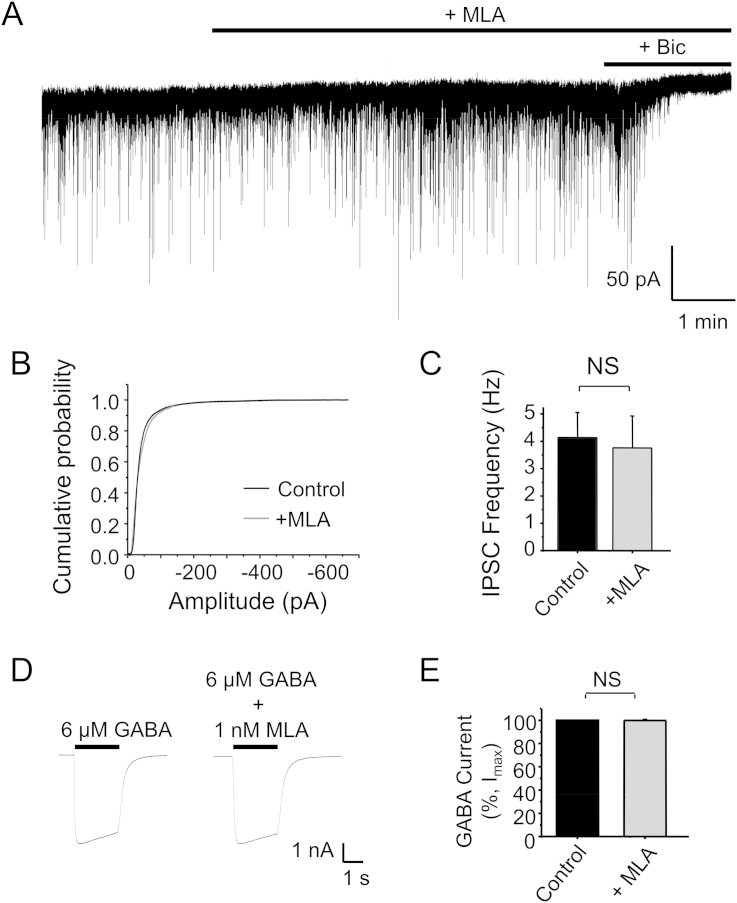
**MLA does not inhibit basal GABA release onto dentate gyrus granule cells**. A, Representative IPSC recording from a dentate gyrus granule cell in control aCSF and after 1 nM MLA. 50 μM Bicuculline (+Bic) was applied at the end of the experiment to block all IPSCs B, Cumulative probability distributions of IPSC amplitudes in control and in 1 nM MLA. C, Frequency of IPSCs in control and in 1 nM MLA. D, Representative whole-cell GABA-activated currents from HEK-293 cells expressing α2β2γ2 receptors, 48 h after transfection, in the absence (left) or presence of 1 nM MLA (right). MLA was pre-applied for 1 min before co-application with GABA. E, Bargraph showing the lack of inhibition of GABA-currents by 1 nM MLA. n = 5–6; NS – not significant.

DGGCs receive inhibitory inputs from local interneurons originating within the dentate gyrus and we initially studied whether any endogenous nAChRs affected GABA release. Although the DGGC holding current was slightly reduced by 1 nM MLA, the frequency of IPSCs remained unaltered (Control: 4.12 ± 0.9 Hz; +MLA: 3.75 ± 1.2 Hz; n = 5; P > 0.05 two-tailed t-test; Fig. 6A, C). The IPSC amplitudes were also unaffected by 1 nM MLA (Control: median IPSC −30.82 pA, n = 5252; +MLA: −30.21 pA, n = 6098; P > 0.05; Fig. 6A–B). Furthermore, in HEK-293 cells, 1 nM MLA did not affect the amplitude of GABA-activated currents of α2β2γ2 receptors (Fig. 6D–E). These results indicated that the release of GABA onto DGGCs from interneurons is not subject to basal control by α7 subunit-containing nAChRs and that MLA does not affect the amplitude of IPSCs. Nevertheless, as a precaution, we included 1 nM MLA in all recording solutions to obviate any α7 nAChR-mediated effects that may have confounded the interpretation of our results.

**Fig. 7:**
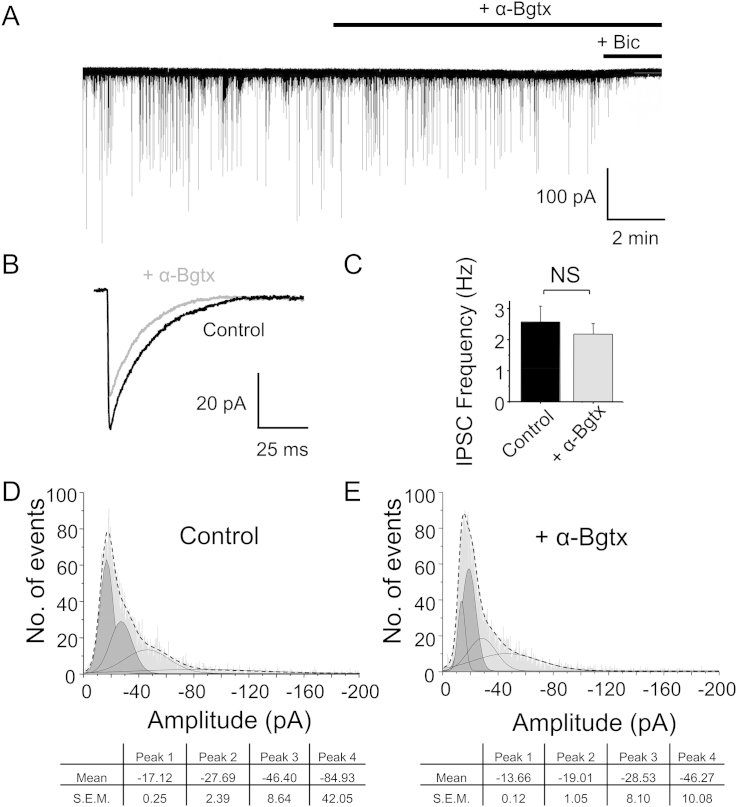
**α-Bgtx inhibits phasic inhibition in hippocampal neurons**. A, Spontaneous IPSCs recorded from adult mouse dentate gyrus granule cells in acute slices (P115–125) in control aCSF and in the presence of 5 μM α-Bgtx (+α-Bgtx). Bicuculline (+Bic, 50 μM) was applied to confirm the GABAergic nature of the postsynaptic currents. Note: 1 nM MLA was present throughout this experiment. B, Averaged IPSCs of 1500 events showing a reduction of amplitudes from (A) in control aCSF (black) or in the presence of α-Bgtx (gray). C, Frequency of IPSCs in control aCSF (black) or in the presence of α-Bgtx (gray). NS – not significant. D, E IPSC amplitude histograms in control aCSF (D) and in the presence of α-Bgtx (E). The parameters for each of the four Gaussians used to obtain optimal fits to the data are shown below. Data in (D) and (E) contain approximately 13000 events.

Then we examined whether α-Bgtx affected GABA release, but the frequency of IPSCs were unaltered by 5 μM α-Bgtx, (Control: 2.57 ± 0.51 Hz, n = 8; P > 0.05; +α-Bgtx: 2.17 ± 0.34 Hz, n = 8, Fig. 7A, C). This also discounted the prospect of other non-α7-containing nAChRs affecting basal release of GABA from interneurons onto DGGCs. However, the median IPSC amplitude was reduced by α-Bgtx (−23.19 pA; n = 6359) compared to control (−26.55 pA; n = 6420) suggesting that α-Bgtx inhibits endogenous synaptic GABA_A_Rs in acute hippocampal slices (Fig. 7B).

The distribution of peak IPSC amplitudes in control and in the presence of α-Bgtx was best described by the sum of four Gaussian components. The mean values for these components in control (Fig. 7D) were reduced by α-Bgtx (Fig. 7E). The leftward shifts of the first (from −17.12 pA to −13.66 pA) and second peaks (from −27.69 pA to −19.01 pA) were significant (<0.001 and P < 0.01, respectively). Although there was a tendency for the two higher means to also be reduced in α-Bgtx, no statistical significance was observed. This may possibly be because they represent currents mediated by receptors predominantly composed non α2-containing receptors that are less sensitive to α-Bgtx.

**Fig. 8:**
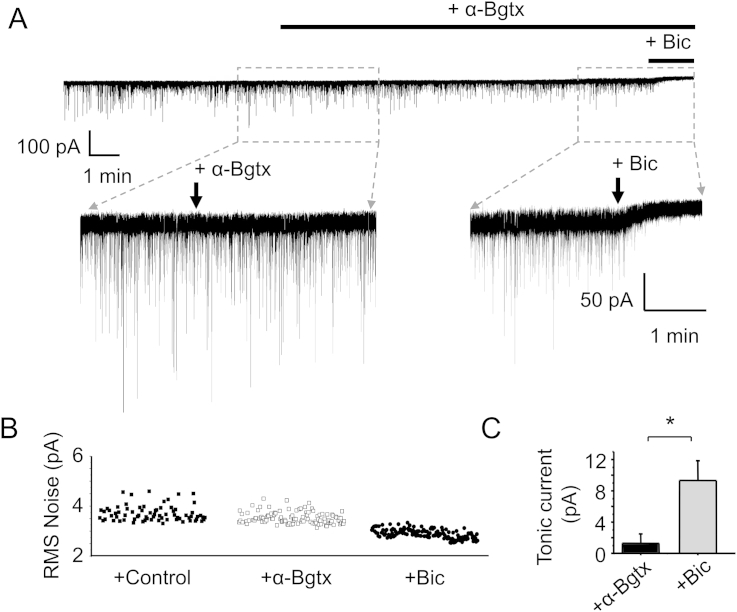
**α-Bgtx and tonic inhibition**. A, Representative IPSCs recorded from dentate gyrus granule cells from acute adult hippocampal slices (P115–125) in control aCSF followed by 5 μM α-Bgtx and 1 nM MLA (+α-Bgtx). Bicuculline (50 μM) was added after α-Bgtx to block GABA_A_Rs and assess the extent of the GABA tonic current. B, Root mean square (RMS) noise from DGGCs in control, and after α-Bgtx and then in bicuculline. RMS noise was only significantly reduced in bicuculline (n = 6, P < 0.05, unpaired two-tailed t-test). C, Net changes in tonic current after application of α-Bgtx or bicuculline.

As α-Bgtx could inhibit postsynaptic GABA_A_Rs, it was plausible that extrasynaptic GABA_A_Rs, which contain β–α interfaces, might also be blocked by α-Bgtx thus affecting tonic inhibition. However, there was no change in RMS noise in the presence (3.39 ± 0.59 pA, n = 6) or absence of 5 μM α-Bgtx (3.55 ± 0.59 pA, n = 6; P > 0.05, two-tailed unpaired t-test; Fig. 8A–B). Application of bicuculline (50 μM) reduced RMS noise significantly (2.74 ± 0.34 pA, n = 6; P < 0.05, Fig. 8A–B) compared to α-Bgtx, indicating the size of the tonic GABAergic current. Similarly, when DGGC holding currents were compared, no change was observed following the application of α-Bgtx (1.27 ± 1.21 pA, n = 7; Fig. 8A, C) although the cells had an average tonic current of 9.31 ± 2.52 pA (n = 7) revealed by applying 50 μM bicuculline. This tonic current was significantly higher than change in holding current observed after the application of α-Bgtx (P < 0.05, Fig. 8C).

To probe the extrasynaptic receptors further, we studied the interaction of recombinant α4β2δ receptors by α-Bgtx in HEK-293 cells, chosen because DGGCs predominantly express α4 and δ subunits, which, most likely, mediate the majority of the tonic inhibition in the DG (Pirker et al., 2000; Stell et al., 2003; Sun et al., 2004).

**Fig. 9:**
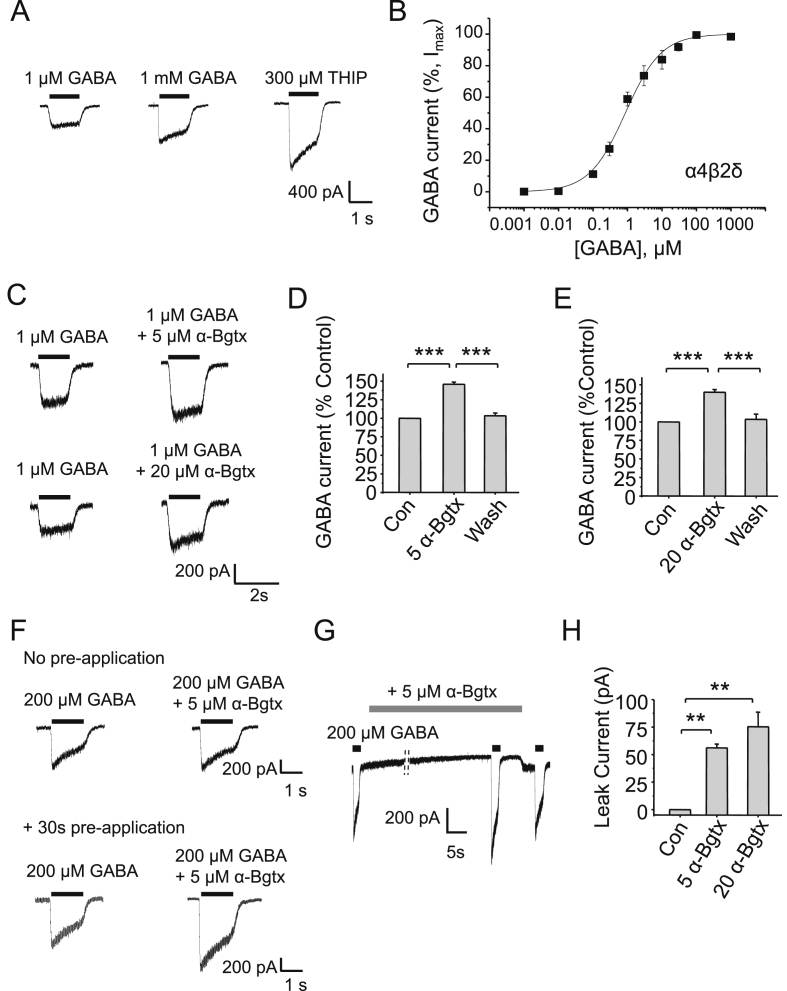
**α-Bgtx does not inhibit GABA-activated α4β2δ currents**. A, Representative whole-cell GABA-activated currents from HEK-293 cells expressing α4β2δ receptors in response to sub-maximal (1 μM) and maximal GABA concentrations (1 mM) in comparison to 300 μM THIP. B, GABA concentration response curve for α4β2δ receptors expressed in HEK-293 cells. Experiments were performed 48 h after transfection. pEC_50_: 6.04 ± 0.12 (n = 5) and EC_50_ = 0.91 μM. C, Representative whole-cell GABA-activated currents in response to submaximal (1 μM) concentrations of GABA in the absence (left-panels) and presence of 5 and 20 μM α-Bgtx (pre-applied for 30s) in HEK-293 cells. D, Potentiation of 1 μM GABA-activated current by 5 μM α-Bgtx compared to control (Con). E, Potentiation of 1 μM GABA-activated current by 20 μM α-Bgtx compared to control (Con). F, Representative whole-cell GABA-activated currents in response to a maximal GABA concentration in the absence (left panel) and presence (right panel) of 5 μM α-BgTx with (bottom panel) or without (top panel) a 30s pre-application of α-BgTx. G, Representative GABA-activated maximal currents before, during, and after application of 5 μM α-Bgtx. Note the change in leak current during α-Bgtx). H, Changes to leak current in control (Con), +5 μM and +20 μM α-Bgtx *P < 0.05, **P < 0.01, ***P < 0.001, n = 3–7, unpaired two-tailed t-test and One-way ANOVA.

To ensure the HEK-293 cells expressed δ subunit-containing receptors, the superagonist ability of THIP (300 μM) at αβδ receptors was confirmed by comparison with responses induced by maximal GABA concentrations (1 mM; Fig. 9A) (Brown et al., 2002; Mortensen et al., 2010). Surprisingly, α-Bgtx potentiated submaximal GABA responses (1 μM; Fig. 9B) at α4β2δ receptors at 5 μM (46 ± 5%, n = 3; p < 0.001; Fig. 9C–D) and 20 μM (40 ± 6%, n = 3; p < 0.001, One-way ANOVA; Fig. 9C, E). This potentiation was observed when α-Bgtx was pre-applied to, but not when co-applied with, GABA (Fig. 9F).

Interestingly, the leak current was clearly reduced by 5 μM (56 ± 3.7 pA, n = 3) and 20 μM α-Bgtx (75 ± 13 pA, n = 3; Fig. 9G–H) when α-Bgtx was pre-applied which may reflect a degree of spontaneous activity for α4β2δ receptors in the absence of GABA similar to that described for α4β3δ receptors (Tang et al., 2010). Pre-application of α-Bgtx may therefore shut spontaneously open α4β2δ receptors. Interestingly though, GABA-activated currents were now potentiated by α-Bgtx, when we would have expected a block similar to that observed with αβγ receptors. Thus α-Bgtx inhibits spontaneously opening α4 and δ subunit-containing receptors but with GABA binding, α-Bgtx is transformed into a potentiator. The mechanism for this is unclear, but may arise by the blocked spontaneously-active channels exhibiting a higher affinity for GABA resulting in a potentiated agonist-activated response. Alternatively, once spontaneity is reduced and GABA is bound, α-Bgtx could bind to another site on the αβδ from which it acts as a positive allosteric modulator. The corollary of this is that α-Bgtx block of spontaneous channels is not equivalent to α-Bgtx block of GABA-activated αβγ receptors.

Given the reduction in the leak current in HEK-293 cells, why do we not see any effect of α-Bgtx on tonic inhibition in neurons? The reduction in the leak current by 5 μm α-Bgtx for HEK-293 cells expressing α4β2δ is over 40-fold greater compared to any reduction by α-Bgtx of the leak in DGGCs. The reasons for this difference may reflect the extent of over-expression of recombinant receptors and differential post-translational processing in cell lines (Tang et al., 2010) that may cause significant levels of spontaneous activity that is not replicated under more physiological conditions. It could also reflect a role for the δ subunit in limiting the binding of α-Bgtx to αβδ receptors in a neuronal setting. If tonic inhibition is mostly due to basal GABA-activated current rather from spontaneously opening receptors in the DGGC, on the basis of the recombinant receptor data, we would expect α-Bgtx to slightly potentiate rather than inhibit tonic current. Overall, these results suggest that α-Bgtx does not inhibit extrasynaptic GABA_A_ receptors, but will inhibit synaptic αxβ2γ2 containing receptors that are most likely populated by the α2β2γ2 isoform.

## 4 Discussion

α-Bgtx is a widely recognised tool for studying the trafficking, expression and inhibition of nAChRs in the nervous system (Changeux et al., 1970; Harel et al., 2001). Its use for imaging has been extended to other receptors, e.g., GABA_A_Rs, by enabling α-Bgtx binding. However, there is little detailed information on whether α-Bgtx could inhibit GABA_A_Rs, though we do know it can affect the function of β3 subunit homomers (McCann et al., 2006). Here, we provide the first report that α-Bgtx can bind to and inhibit recombinant and native neuronal GABA_A_Rs in a mixed inhibitory manner.

Our findings serve as a cautionary note. Firstly, using α-Bgtx to study nAChR function in the nervous system will not be specific unless attention is paid to the concentrations used. Secondly, the use of α-Bgtx as a tool to study receptor trafficking gained popularity because a 13-amino acid mimotope (WRYYESSLEPYPD; Harel et al., 2001) forms a high affinity α-Bgtx binding site (BBS) and this can be introduced into receptors relatively easily, enabling α-Bgtx binding. The BBS has been engineered into: GluA2 AMPA receptor (Sekine-Aizawa and Huganir, 2004), GABA_A_R α2 (Brady et al., 2014), β3 (Bogdanov et al., 2006; Saliba et al., 2007), γ2 (Joshi et al., 2013), and δ (Joshi et al., 2013) subunits: GABA_B_ R1a (Hannan et al., 2011), R1b (Hannan et al., 2012) and R2 subunits (Hannan et al., 2011); mGluR2 (Hannan et al., 2012); Kv4.2 channels (Moise et al., 2010), and Ca^2+^ channel α_2_δ-2 subunits (Tran-Van-Minh and Dolphin, 2010). This approach has greatly improved our understanding of receptor trafficking and expression. However, for imaging studies with fluorophore-linked α-Bgtx, its ability to bind to native GABA_A_Rs could complicate the interpretation of results.

Several studies have employed strategies to avoid problems associated with α-Bgtx binding to principally nAChRs. For example, AMPAR (Sekine-Aizawa and Huganir, 2004), GABA_A_R (Brady et al., 2014) and GABA_B_R (Hannan et al., 2011, 2012) trafficking studies have used d-Tc to prevent binding of α-Bgtx to nAChRs. From our and other studies, it is clear that high doses of d-Tc can prevent α-Bgtx binding to GABA_A_Rs, but also cause direct inhibition of GABA-activated currents. Another strategy to avoid complications would be to limit the over-expression of GABA_A_Rs by using a controlled transfection of only specific subunits (Joshi et al., 2013). In this regard, over-expression of β3 subunits could result in the formation of homomers with β3–β3 interfaces, which bind to α-Bgtx without the need for an engineered BBS.

In addition, low concentrations of fluorophore-conjugated α-Bgtx can also be used to avoid labelling of GABA_A_Rs as the affinity of α-Bgtx for endogenous GABA_A_Rs is lower compared to that for the BBS. Under our experimental conditions, we do not observe any binding of fluorescent α-Bgtx (400 nM) during live cell confocal microscopy to E18 rat hippocampal cultured neurons. These cultures express very low levels of α7 nAChRs and robust staining with α-Bgtx can only be observed at this concentration (400 nM) when the neurons are transfected with membrane expressing BBS-tagged receptors. Similarly, the over-expression of recombinant receptors in heterologous expression systems (HEK-293 cells) allows the detection of α-Bgtx fluorescence at low concentrations.

Although pentameric ligand-gated ion channel family members share many common structural features, they exhibit relatively distinct pharmacological profiles. However, there are circumstances where some ligands can affect more than receptor type, notably GABA_A_R β3 homomers binding of α-Bgtx (McCann et al., 2006). In the present study, we extend this observation to α-Bgtx inhibiting physiologically-important GABA_A_R heteromers. In addition to α7 homomers, α-Bgtx also inhibits αβγδ or αβδε heteromeric nAChRs. This suggests that the α-Bgtx binding site on heteromeric GABA_A_Rs may share a similar architecture to the binding site found on heteromeric nAChRs whereas the β3 α-Bgtx binding could be similar to homomeric nAChRs. The β3-homomeric receptors are a pharmacologically distinct population of GABA_A_Rs that do not respond to GABA, but are sensitive to potentiation by bicuculline (Wooltorton et al., 1997). Therefore, the α-Bgtx binding site on these homomers is likely to be different from binding site on αβγ heteromers. For muscle-type nAChRs, α-Bgtx binding occurs at the interface between α-γ and α-δ subunits. From our study, the two β–α interfaces of αβγ GABA_A_Rs (which are comparable to the α-γ, α-δ nAChR interfaces (Smart and Paoletti, 2012)), could therefore contain a similar α-Bgtx binding site. This deduction is based upon the abolition of fluorophore-linked α-Bgtx binding to GABA_A_Rs by competitive GABA receptor antagonists. This is in accord with α-Bgtx binding at the GABA-binding β–α interface, enabling α-Bgtx to be used as a tool to study GABA_A_R function. However, for α4β2δ receptors, GABA responses were not inhibited by α-Bgtx. This was surprising since these receptors will retain the β–α interfaces, so we must presume the fifth subunit (δ) does have some influence on whether α-Bgtx will bind and cause inhibition.

Profiling α-Bgtx binding (by fluorescence) and inhibition (by electrophysiology) at low concentrations demonstrated that the highest levels of both were achieved with α2β2γ2 receptors. This preference for α2β2γ2 is likely to reflect different binding affinities of α-Bgtx for various β2-αx interfaces, presumably because of divergent amino acid sequences between the different α subunits on the complementary (−) (as opposed to the principal side (+)) side of the β–α subunit interface (Smart and Paoletti, 2012). From the fluorescent α-Bgtx binding profiles of αxβ1γ2 receptors, we would expect the α-Bgtx inhibitory profiles to be similar, with α2β1γ2 being inhibited most and α5β1γ2 least, by α-Bgtx.

The receptor subtype selectivity of α-Bgtx is useful for studying native GABA_A_R function in acute hippocampal slices, without any dependence upon nAChRs. Application of MLA revealed that presynaptic α7-containing nAChRs are not regulating the basal release of GABA onto adult DGGCs. However, previous reports suggest that nAChR-activation can cause increased GABA release (Radcliffe et al., 1999), though, MLA had no effect on basal GABA release in mouse CA1 pyramidal neurons (Proctor et al., 2011) and layer V prefrontal cortex (Aracri et al., 2010). In addition, α-Bgtx neither affected the average tonic current nor the RMS noise suggesting that other subtypes of nAChRs do not control basal GABA release in the dentate. This further suggested that α-Bgtx, at concentrations specific for inhibiting α2 subunit-containing receptors, has little if any effect on α4 or α5 subunit-containing extrasynaptic receptors.

The inhibitory effect of α-Bgtx on IPSC amplitudes in adult slices was rapid in onset. The reduced IPSC amplitudes were more pronounced for smaller compared to larger events, and given the selectivity of α-Bgtx, implies that α2β1/2γ2 receptors contribute largely to these sub-populations of events. Of course, a small proportion of IPSCs may be inhibited by the binding of α-Bgtx to β3 subunit-containing receptors. Nevertheless, the binding of α-Bgtx to heteromeric GABA_A_Rs together with the inhibition of GABA-activated currents in HEK-293 cells, primary hippocampal neurons and in slices, clearly indicates that α-Bgtx can act as an antagonist at native GABA_A_Rs to block synaptic inhibition, presumably by binding at the GABA_A_ receptor β–α interface.

## Author contribution

SH carried out the imaging and electrophysiology of slices. MM and SH carried out the electrophysiology of recombinant receptors and cultured neurons. SH and TGS designed the project and wrote the paper. All authors contributed to the writing of the paper.
